# CRB1 Gene Mutation Causing Different Phenotypes of Leber Congenital Amaurosis in Siblings

**DOI:** 10.18502/jovr.v14i4.5467

**Published:** 2019-10-24

**Authors:** Shaheryar Ahmed Khan, Achim Richard Nestel

**Affiliations:** ^1^North Devon District Hospital, Barnstaple, Devon, UK

**Keywords:** *CRB1*, LCA, Retinitis Pigmentosa, Rod cone Dystrophy

## Abstract

**Purpose:**

We report a rare case of *CRB1*gene mutation in two siblings (sisters) affected with the exact same genetic mutation on both *CRB1*genes resulting in varying phenotypes.

**Case Report:**

*CRB1*gene mutation in this case has resulted in causing varying degrees of Leber congenital amaurosis (LCA) in both sisters with a more severe phenotype in the older sibling causing LCA-8 with retinitis pigmentosa spectrum in both eyes and a milder phenotype causing LCA-8 with less severe rod cone dystrophy in the younger sister.

**Conclusion:**

In summary, the mechanisms of varying phenotypes resulting from *CRB1* genetic mutation are still not well understood. We concluded that the presence of different phenotypes associated with identical genotypic mutation of a single gene in siblings or in a family is important especially when dealing with retinal dystrophies.

##  INTRODUCTION

Mutations in *CRB1*gene are associated with several types of autosomal recessive retinal dystrophies, such as retinitis pigmentosa, Leber congenital amaurosis (LCA),^[1]^ and Coats-like vasculopathy.^[1]^

LCA is the most common genetically defined, severe rod cone dystrophy causing pronounced visual impairment. It has a very guarded prognosis and usually progresses very rapidly but could be even worse when associated with a complication such as Coats-like vasculopathy.^[1]^ LCA has been associated with 18 different genetic mutations.^[2]^ *CRB1*gene mutation is responsible for causing LCA defined as type 8.^[2,3]^ Our case report represents two sisters affected by exactly identical *CRB1*gene mutation but exhibiting varying phenotypes that have not been reported previously for this disease spectrum in siblings (sisters).

##  CASE REPORT

We present two sisters affected by *CRB1*gene mutation.

###  First Patient (Older Sister)

A 58-year-old female patient visited the retina clinic in the North Devon District Hospital (NDDH), Barnstaple, UK in April 2012. She had a long history of poor vision and complained of further deterioration of vision in both eyes; she was referred to the clinic by her general practitioner (GP).

The patient had suffered from significant ocular problems since childhood. The condition progressed rapidly, and she was registered as blind after a few years. She was previously diagnosed with severe macular dystrophy in both eyes at the Moorfields Eye Hospital, London. A differential diagnosis suggested a possibility of fundus flavimaculatus, Sorsby's dystrophy, and Best vitelliform macular dystrophy. The patient underwent fluorescein angiography at the Moorfields Eye Hospital which revealed profound atrophy of retinal pigment epithelium (RPE) at the posterior pole in both eyes along with widespread retinal edema and microvascular changes. She also underwent electrophysiological testing at that time, which included electroretinogram (ERG) and electrooculogram (EOG). ERG and EOG were both performed on an in-house manufactured electrophysiological visual evoked potential machine called the “Observe-reviewer” system at the Moorfields Eye Hospital, UK. The EOG was reported to be abnormal and showed no light rise in either eye. The ERG was performed with gold foil recording electrodes according to the international standards. It showed very low scotopic and photopic responses on both sides. Her rod ERG showed almost no response and there were very small “a” and “b” waves which were markedly delayed on both sides. The single flash cone ERG response also had a very small amplitude with significantly delayed latency and the single flash cone ERG “b” wave amplitude was recorded to be less than the half of a normal response. In summary, her full-field ERG was abnormal for rods and cones, both being very reduced and delayed.

Her family history revealed unknown eye problems in her younger sister as well.

**Figure 1:**
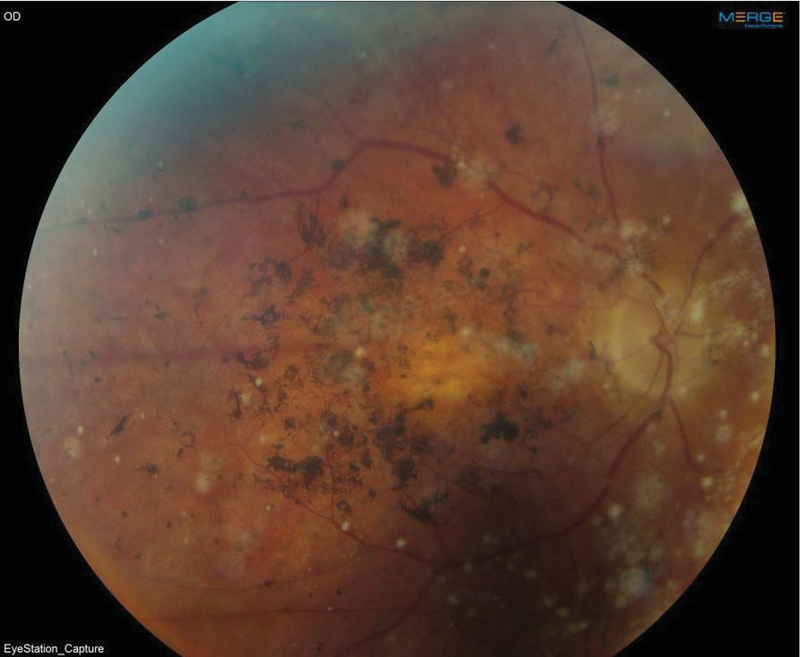
Right fundus picture of older sister showing bony spicule pigmentation and foveal atrophy.

**Figure 2:**
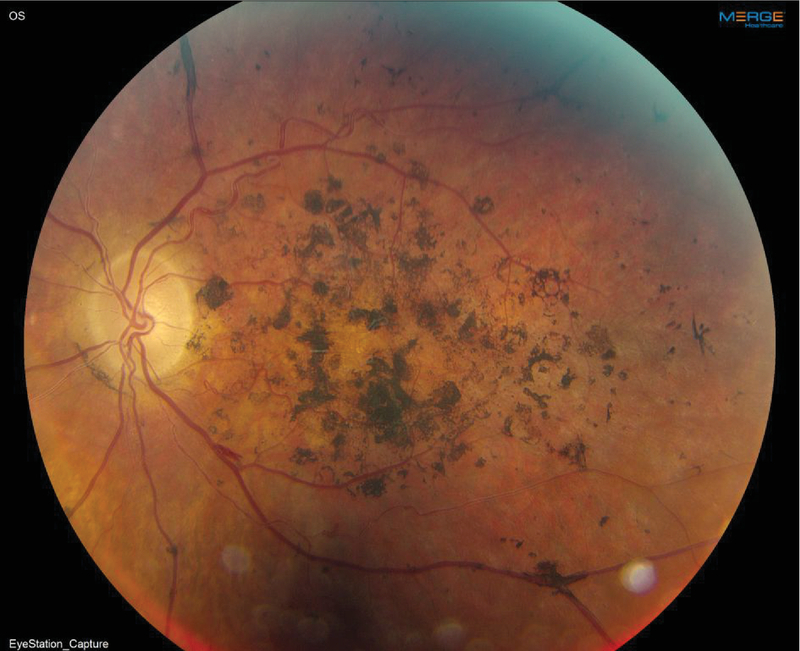
Left fundus picture of older sister showing bony spicule pigmentation and foveal atrophy.

**Figure 3:**
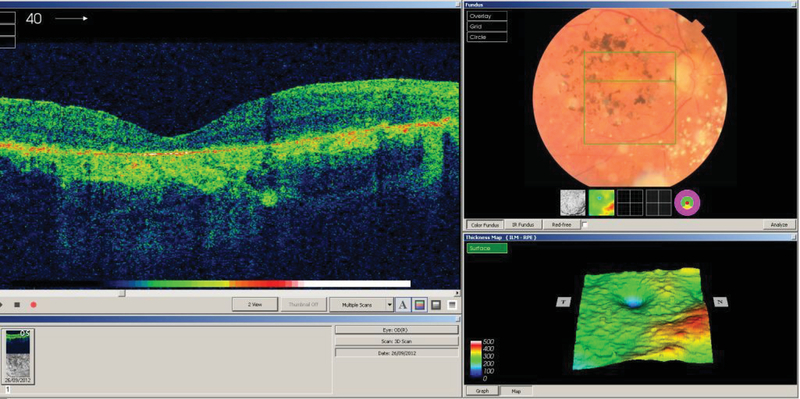
Right OCT of older sister showing hypertrophy of RPE layer and thinning of outer retinal layers.

On examination in the clinic at NDDH, the woman could count fingers using both eyes and the vision did not improve with pinhole examination. She had an unremarkable anterior segment examination with clear lenses. Pupil reactions were sluggish on both sides. Intraocular pressure (IOP) was in the normal range in both eyes. Retinal examination revealed retinal pigmentation with some bony spicules mainly affecting her macular areas along with paravenous pigmentation seen in some areas of both eyes as observed from her fundus photos [Figures 1 and 2]. Macular examination did not show signs of macular edema. She had mildly pale optic discs bilaterally. Optical coherence tomography (OCT) scan was performed with Topcon 3D-OCT 2000 series model (Topcon Corporation, Tokyo, Japan) which showed outer retinal layer atrophy along with pigmentation in the macula and no macular edema bilaterally [Figure 3].

The patient had a genetic testing for her eye condition at the closest genetic testing center in 2012 on a specialist's advice. The molecular and genetic testing results were received in 2014, and the reports showed that she carried a compound heterogenous pair of alterations in the *CRB1*gene that is associated with RP (type 12) and LCA type-8 due to the mutation. The geneticist reported that more appropriate designation is LCA-8 as one of the alleles is a null allele which is more frequently associated with LCA type-8. The methodology used for genetic testing was a next-generation massively parallel sequencing with a panel of 105 genes associated with retinal dystrophy. The abnormal findings were then confirmed through Sanger sequencing. The patient's detailed results for both *CRB1*genes having mutations were *CRB1*c.498_506del9 p.(IIe 167_Gly169del) and *CRB1*c.2688T>A p.(Cys896Ter). She was then identified as harboring *CRB1*gene mutation causing LCA type-8 with retinitis pigmentosa spectrum in both eyes.

###  Second Patient (Younger Sister)

A 53-year-old lady was examined in the eye clinic at the NDDH in September 2015 as referred by her GP as she had problems with her right eye vision since a young age like her older sister. She also underwent a genetic testing several months ago. She was initially diagnosed as having probable age-related macular degeneration based on clinical examination at the Moorfields Eye Hospital. She also had an ERG (done with Observer-reviewer system machine at the Moorfields Hospital, UK) which was performed with gold foil recording electrodes according to the international standards and was found to be subnormal on the right side with lower level responses. Rod-specific ERG-b wave amplitudes were 145 µV on the right and 270 µV on the left sides, respectively. Bright flash ERG “a” and “b” wave amplitudes were 170 µV and 280 µV in the right eye and 305 µV and 560 µV in the left eye, respectively. She also had a pattern ERG P50 which was also found to be subnormal (0.6 µV) on the right side being reported as a delayed pattern ERG. The EOG (also done with Observer-reviewer system machine at the Moorfields Hospital, UK) was essentially normal for both eyes. The EOG light rise was 185% in the right eye and 200% in the left eye.

**Figure 4:**
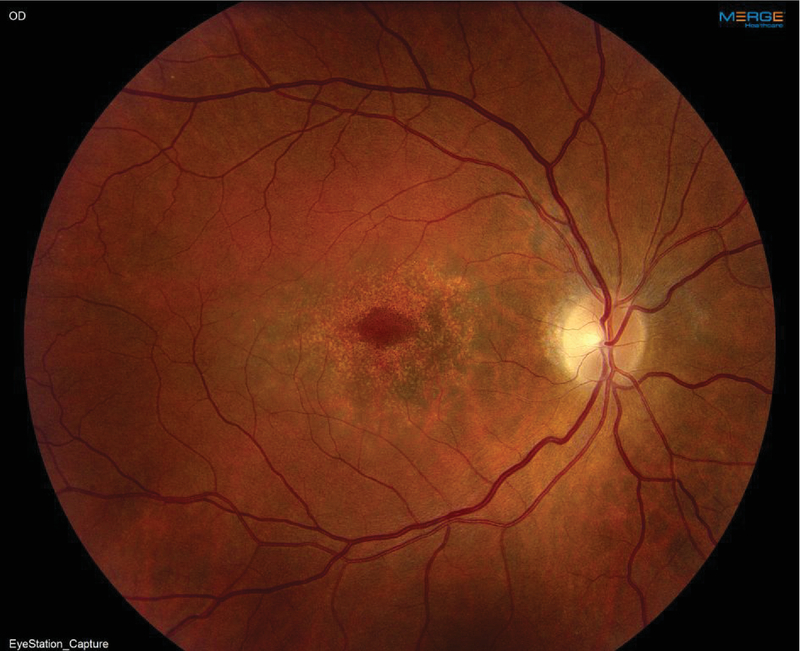
Right fundus picture of younger sister showing central macular atrophy with a mottled fundus appearance.

**Figure 5:**
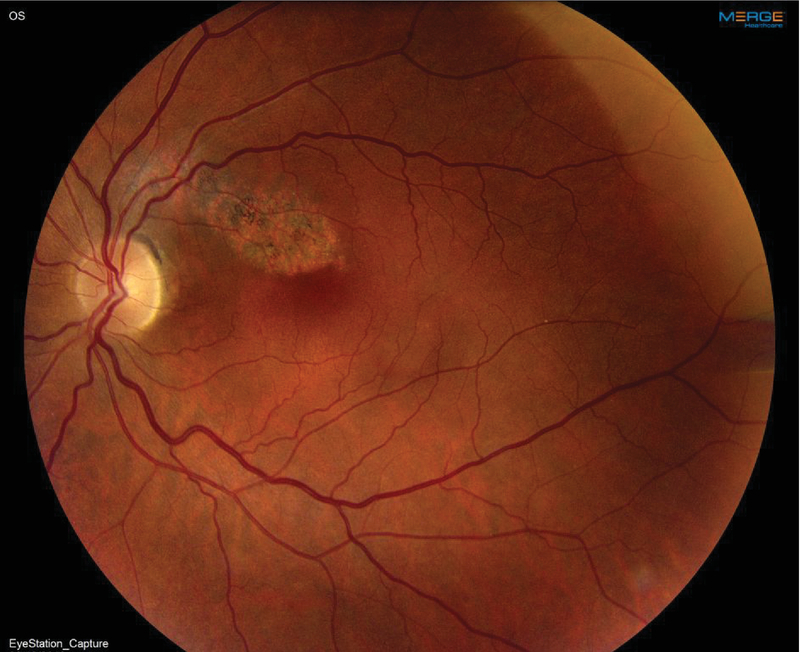
Left fundus picture of younger sister showing eccentric flat atrophic patch sparing the macula.

**Figure 6:**
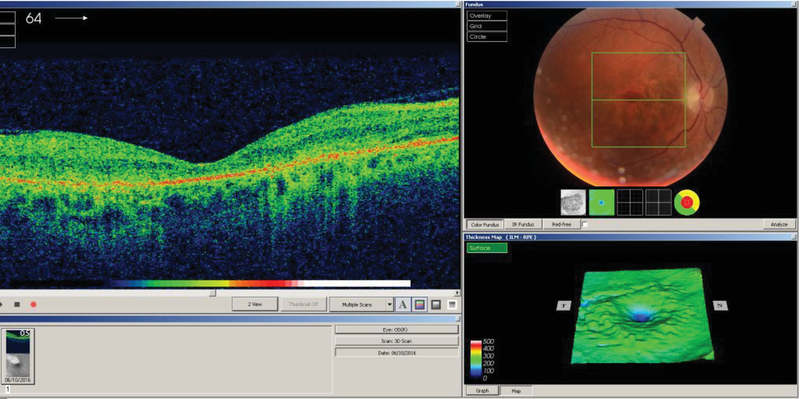
Right eye OCT scan of younger sister showing thinning of outer retinal layers.

The patient complained of poor vision in her right eye. On examination in the eye clinic, the best corrected visual acuity was 6/36 in the right and 6/6 in the left eye. Anterior segment examination was normal and unremarkable. IOP was in normal range on both sides. On dilated funduscopy, a central macular atrophy with a mottled fundus appearance on the right side and an eccentric atrophic patch on the left side, just above the macular area, sparing the macula, were observed which were also visible in the fundus photos [Figures 4 and 5]. Optic discs were found to be normal on both sides. An OCT scan was performed with Topcon 3D-OCT 2000 model which revealed RPE atrophy in the right eye along with thinning of the outer retinal layers [Figure 6] and a less marked foveal sparing RPE atrophic patch in the left eye. The genetic testing in her case was done through Sanger sequencing, like her sister. Her genetic testing results showed a compound heterozygous genetic mutation of both *CRB1*genes which was exactly similar to her older sister. Her detailed results for both *CRB1*genes having mutations are *CRB1*c.498_506del9 p.(IIe 167_Gly169del) and *CRB1*c.2688T>A p.(Cys896Ter). Geneticist confirmed that the mutations were affecting both alleles of the *CRB1*gene and since both of them were nonsense mutations, they were securely pathogenic and not carrier genes; this causes LCA-8. Hence, she was considered to have a milder phenotype of LCA type-8.

##  DISCUSSION 

*CRB1*is a human homologue of the Drosophila melanogaster gene crumbs (crb) and is localized in the inner segment of mammalian photoreceptors and the human fetal brain.^[4]^ The *CRB1*gene is located on chromosome 1q31.3 in humans and is composed of 12 exons generating two different transcripts of 1376 and 1406 amino acids. It plays a crucial role in photoreceptor morphogenesis and subsequent function. *CRB1*has been indicated to be involved in mechanisms that control intracellular communication and polarity as well as maintain adherent junctions of epithelial cells.^[5,6]^

*CRB1*gene mutation is one of the most common genetic mutations found in the LCA patients. According to some studies, about 7–10% of LCA patients^[7,8]^ have *CRB1*gene mutation as the underlying cause.

*CRB1*gene mutation is also responsible for a characteristic form of autosomal recessive RP which is known as RP12.^[9]^ Unfortunately LCA-8 has no treatment at present. Only one type of LCA that is type-2 has been successfully treated using gene therapy in 2008.^[10,11]^

*CRB1*gene mutation results in many phenotypes. In the studies conducted till date, there has been no clear or defining genotype–phenotype relationship established in *CRB1* disease.^[12]^ One meta-analysis study suggested that different phenotypes of patients with *CRB1* gene mutations might be due to additional modifying factors (genetic or/and environmental) rather than a particular mutant allele combination.^[13]^

Variations in the coding sequence of the *CRB1* gene mutation can cause disease, resulting in different phenotypes according to some researchers.^[14]^

Li et al^[15]^ have described the association of *CRB1* mutations with RP and LCA suggestive of early onset RP being a spectrum from LCA to RP and genes associated with early onset RP being potentially good candidates for causing LCA and vice versa.

*CRB1* gene mutation in this case has resulted in causing varying degrees of LCA-8 in both sisters with a severe phenotype in the older sibling (LCA-8 with retinitis pigmentosa spectrum in both eyes) and a milder phenotype causing LCA-8 with less severe rod cone dystrophy in the younger one. It is interesting to note that both sisters have similar genotype and exactly same set of mutations but they exhibit varying phenotypes. In summary, mechanisms of *CRB1* genetic mutation leading to varying phenotypes are still not well understood. Our case report is rare and interesting in the fact that we are presenting a case, which to the best of our knowledge, has not been reported previously in siblings for such scenarios where the exact same *CRB1* genotypic mutation in two sisters resulted in markedly different phenotypes. We further concluded that the presence of different phenotypes associated with identical genotypic mutations in the same gene in siblings or in a family must be considered especially when dealing with retinal dystrophies.

##  Financial Support and Sponsorship

Nil.

##  Conflicts of Interest

There are no conflicts of interest.
